# Evidence for the important role of inflammation in xenotransplantation

**DOI:** 10.1186/s12950-019-0213-3

**Published:** 2019-05-28

**Authors:** Juan Li, Hidetaka Hara, Yi Wang, Charles Esmon, David K. C. Cooper, Hayato Iwase

**Affiliations:** 1 Second Affiliated Hospital, University of South China, Hengyang City, Hunan China; 2 Xenotransplantation Program, Department of Surgery, University of Alabama at Birmingham, Birmingham, AL USA; 3 Coagulation Biology Laboratory, Oklahoma Medical Research Foundation, Oklahoma City, OK USA

**Keywords:** Inflammation, Non-human primates, Pigs, Xenotransplantation

## Abstract

There is increasing evidence of a sustained state of systemic inflammation after pig-to-nonhuman primate (NHP) xenotransplantation (that has been termed systemic inflammation in xenograft recipients [SIXR]). Increases in inflammatory markers, e.g., C-reactive protein, histones, serum amyloid A, D-dimer, cytokines, chemokines, and a decrease in free triiodothyronine, have been demonstrated in the recipient NHPs. The complex interactions between inflammation, coagulation, and the immune response are well-recognized, but the role of inflammation in xenograft recipients is not fully understood. The evidence suggests that inflammation can promote the activation of coagulation and the adaptive immune response, but the exact mechanisms remain uncertain. If prolonged xenograft survival is to be achieved, anti-inflammatory strategies (e.g., the administration of anti-inflammatory agents, and/or the generation of genetically-engineered organ-source pigs that are protected from the effect of inflammation) may be necessary to prevent, control, or negate the effect of the systemic inflammation that develops in xenograft recipients. This may allow for a reduction in the intensity of exogenous immunosuppressive therapy. If immunological tolerance to a xenograft is to be obtained, then control of inflammation may be essential.

## Introduction

Organ transplantation is one of the medical success stories of the past 70 years, but there remain insufficient organs from deceased human donors to treat all of the patients who might benefit. For example, in the USA at present there are approximately 120,000 patients awaiting an organ of one sort or another, and yet this year only approximately 10,000 deceased human donors will become available, providing an average of three or four organs per donor [1].

The lack of human organs could be obviated if a suitable animal source of organs were available. For a number of logistic and other reasons, the pig has been identified as a potential source of organs for clinical transplantation [2]. The field of xenotransplantation (cross-species transplantation) has therefore been extensively investigated during the past 35 years [3]. Although organs from wild-type (i.e., genetically-*un*modified) pigs transplanted into humans or nonhuman primates (NHPs) are rejected within minutes [4], our ability to genetically-engineer the pig to protect its organs from the primate immune response has resulted in life-supporting kidney or heart graft survival in NHPs extending to many months or even more than a year [5–9]. One of the barriers that has had to be overcome, but continues to be problematic, is the inflammatory response to the presence of a pig organ.

Inflammation is part of the complex biological response of body tissues to harmful stimuli, and is observed in various diseases, e.g., inflammatory disease [10], infection [11], atherosclerosis [12]. The release of appropriate pro-inflammatory cytokines and chemokines is necessary for protective immunity, but production of these factors in excess can result in various pathological states [13]. An inflammatory response follows ischemia-reperfusion injury after organ transplantation [14]. This may play an important role in initiating the allo-immune response [15], and in the development of allograft vasculopathy [16].

There is increasing evidence of a systemic inflammatory response to the presence of a pig xenograft (‘systemic inflammation in xenograft recipients’ [SIXR]) [17–19]. Inflammation promotes activation of coagulation [17–21] and of the immune response [17, 18] that develop after xenotransplantation [22, 23]. In organ xenograft recipients, C-reactive protein (C-RP) increases *before* the development of consumptive coagulopathy or a T cell response [17, 18]. Infiltrating innate immune cells express tissue factor, which plays a role in initiating coagulation [24]. The development of T cell tolerance is inhibited by inflammation [22, 25].

We here review the evidence of a prolonged systemic inflammatory response to a xenograft, and consider what steps can be taken to prevent or reduce it. We have primarily drawn on our own observations, but have supplemented these by a review of the literature.

### Evidence for a sustained inflammatory response in xenograft recipients (SIXR) (Table 1)

*C-reactive protein (C-RP)* is an acute phase protein synthesized largely by hepatocytes in response to proinflammatory cytokines, in particular interleukin-6 (IL-6) [31]. C-RP provides the first line of defense to an invasive pathogen, and can promote activation of complement, bacterial capsular swelling, and phagocytosis [32]. It is a marker of early infection, and provides an easy objective parameter [33]. Moreover, C-RP mRNA expression increases in the presence of acute rejection of a renal allograft [34]. C-RP can contribute both to host defense against infection and enhancement of inflammatory tissue damage.

**Table 1:** Evidence for systemic inflammation in xenograft recipients (SIXR)

	Indicators of inflammation	Change when associated with xenotransplantation	References
In vivo	C-reactive protein (C-RP)	↑	[13, 19, 26]
	Serum amyloid A (SAA)	↑	[26–28]
	Histones	↑	[26]
	D-dimer	↑	[6, 13, 19]
	Tumor necrosis factor-alpha (TNF-α)	↑	[18]
	Interferon-gamma (IFN-γ)	↑	[18]
	Interleukin-6 (IL-6)	↑	[13, 18]
	Interleukin-8 (IL-8)	↑	[13, 18]
	Interleukin-12 (IL-12)	↑	[18]
	Monocyte chemotactic protein-1 (MCP-1)	↑	[13, 18]
	Soluble CD40 ligand (sCD40L)	↑	[13, 29]
	Free triiodothyronine (fT3)	↓	[6, 30]
In vitro	Platelet aggregation	↑	[26]
	Endothelial cell apoptosis	↑	[26]

**Fig. 1:**
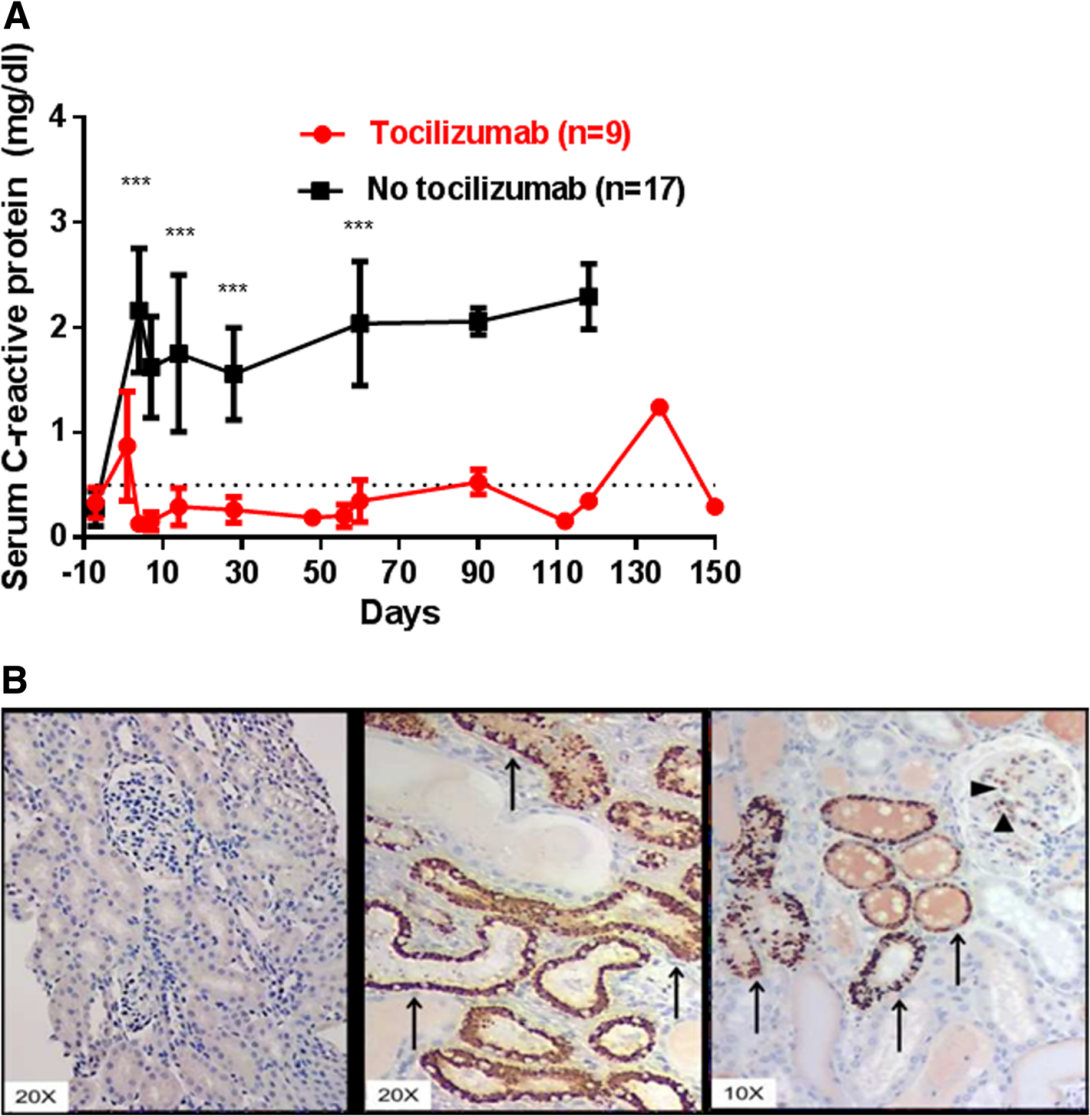
**a** C-RP in baboons with pig artery patch (*n* = 9) or organ (*n* = 17) grafts. Levels of C-RP in baboons before (day 0) and after pig organ or artery patch transplantation. (Black line = without tocilizumab therapy; Red line = with tocilizumab therapy.) The mean level of C-RP in the tocilizumab-treated baboons remained < 0.5 mg/dL from day 4, which (on days 7, 14, 28, and 60) was significantly lower than in baboons not receiving tocilizumab (day 7, 0.2 vs 1.6 mg/dL, *P* < 0.001; day 14, 0.3 vs 1.8 mg/dL, P < 0.001; day 28, 0.3 vs 1.6 mg/dL, P < 0.001; and day 60, 0.3 vs 2.0 mg/dL, *P* < 0.01, respectively). (**P < 0.01; ***P < 0.001). Tocilizumab treatment therefore prevented an increase in C-RP after xenotransplantation. (The rise in C-RP on day 136 in one of the baboons in the tocilizumab-treatment group was associated with the onset of systemic infection.) (Reprinted with permission from ref. [26]). **b** C-RP deposition in pig kidneys transplanted into baboons, an indicator of the inflammatory response to the graft. (Left panel) At 30 min after reperfusion of an α1,3-galactosyltransferase gene-knockout (GTKO) pig kidney graft, no C-RP deposition was detected. In two different kidneys at the time of euthanasia (middle and right panels), C-RP deposition was detected in the glomeruli (arrow heads, right panel) and tubules (arrows, middle and right panels). Our data suggest that both the xenograft and the recipient contribute to C-RP production. (We detected minimal C-RP in NHPs undergoing heart *allo*transplantation [not shown]). (Reproduced with permission from ref. [18])

After pig-to-baboon organ transplantation, C-RP is increased for several months, suggesting a persisting inflammatory state [13, 19, 26] (Fig. 1a), and is deposited in the transplanted pig kidney [18] (Fig. 1b). Whether this is secondary to initial antibody binding remains uncertain.

*Serum amyloid A (SAA)* is a major acute-phase protein and an inflammation-related marker in tuberculosis, rheumatoid arthritis, Crohn’s disease, and in various cancers [35, 36]. SAA is also a sensitive marker of acute allograft rejection [37]. Hepatocytes are a major source of SAA [38]. Elevated SAA results from increases in circulating serum interleukin-6 (IL-6) and tumor necrosis factor-alpha (TNF-α) [39]. The inflammation-associated cytokines produced by endothelial cells (ECs), lymphocytes, specially-activated monocytes, and macrophages stimulate amyloid A synthesis [35, 40]. In turn, SAA may induce the release of some pro-inflammatory cytokines e.g., TNF-α, IL-1β, and the chemokine IL-8 [41, 42]. However, SAA can also induce the secretion of chemokines that might suppress inflammation locally [43], and mobilizes phospholipids and cholesterol for cell repair [44].

**Fig. 2:**
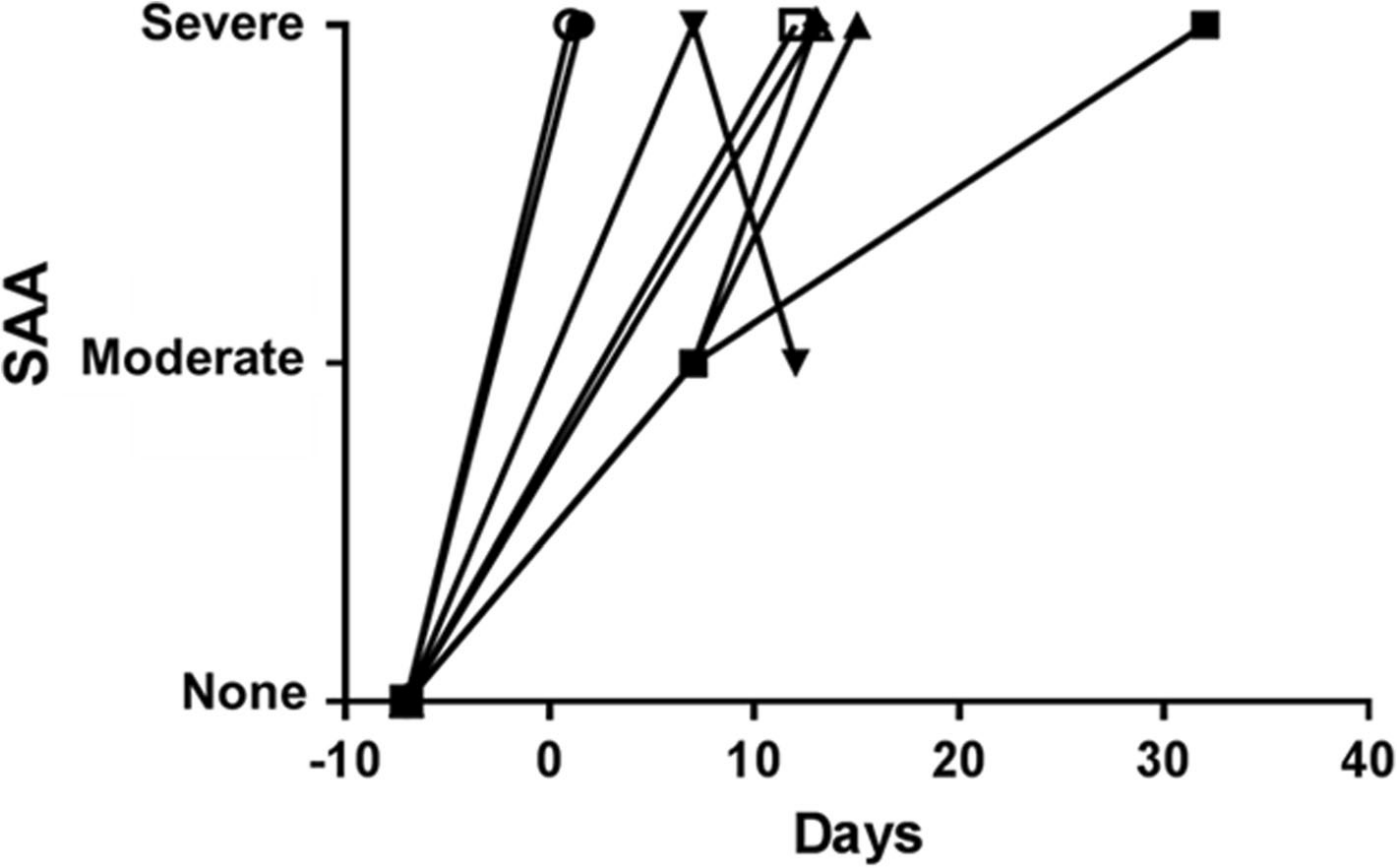
Serum amyloid A (SAA) in baboons with pig kidney grafts that failed within the first post-transplant month. The SAA increased immediately after pig kidney transplantation, and never returned to pre-transplant levels. Other measurements indicated that a state of inflammation had developed

After pig-to-baboon organ xenotransplantation, significant increases in SAA have been observed during antibody-mediated rejection (Fig. 2) or when a consumptive coagulopathy or infection is developing [26, 27]. Amyloid A is deposited in the transplanted pig kidney [28]. Although the current method of measuring SAA is not fully quantitative, it is a simple and rapid indicator of the inflammatory state, allowing early investigation, e.g., for rejection, infection, or other complications.

*Extracellular histones* play a key role in inflammation [45]. In vivo, they result in EC dysfunction (e.g., neutrophil margination, hemorrhage, thrombosis), and in vitro they are cytotoxic to ECs [45]. Five types of histones have been identified [46, 47]. Release of histones can be triggered by sepsis, trauma, chemical toxicity, transplant injury, and ischemia-reperfusion [48]. They bind to Toll-like receptors (TLRs) of various cells, e.g., platelets, red blood cells [49], which in turn induce NETosis (cell death, release of granular contents into the extracellular space). This in turn increases histone release and amplifies inflammation [50–57].

**Fig.3:**
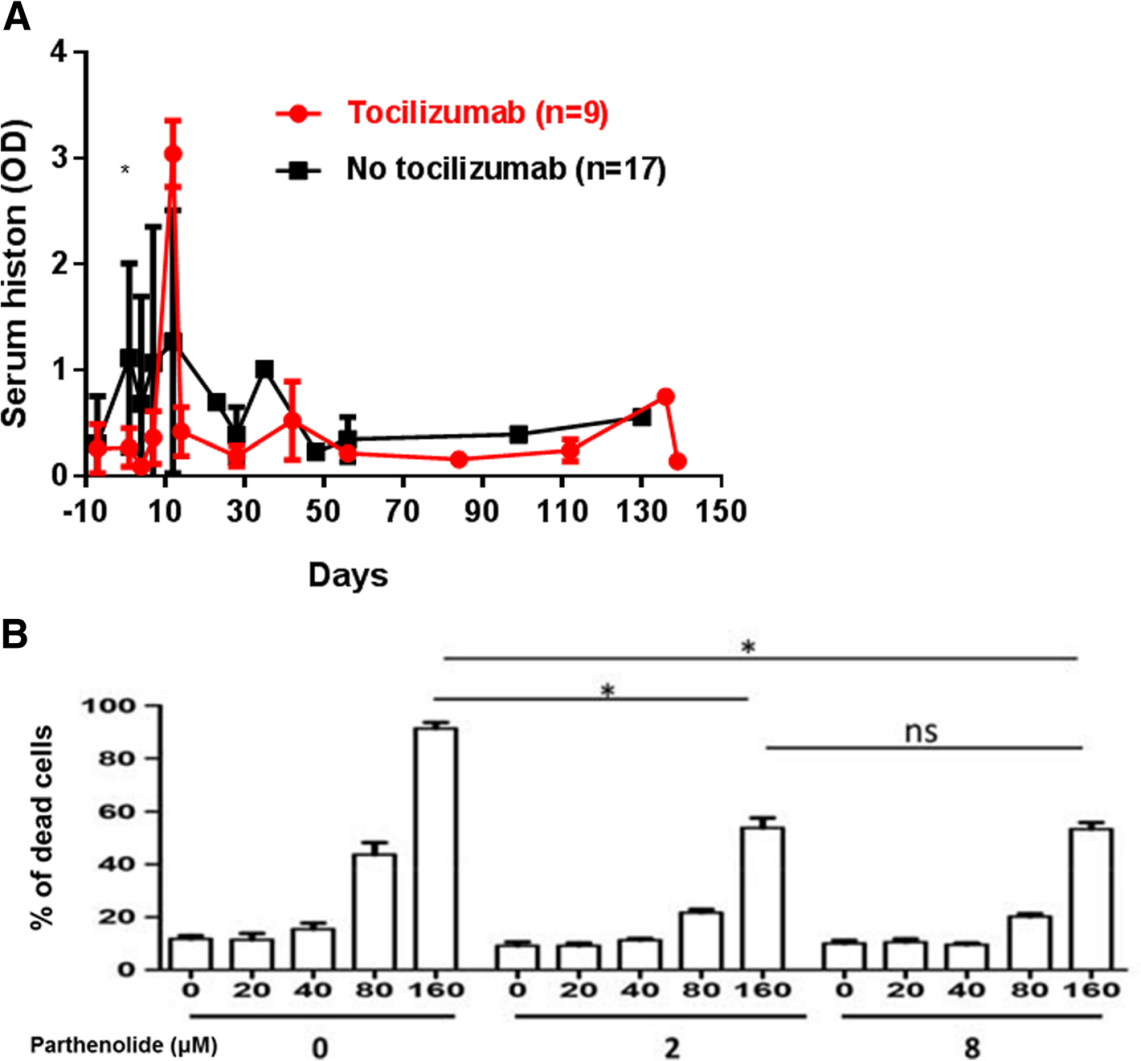
**a** Serum extracellular histone levels in baboons with pig artery patch grafts. In the absence of tocilizumab therapy, the mean serum histone level was higher on post-transplant day 1 than in baboons receiving tocilizumab (day 1, 1.2 vs. 0.3, *P < 0.05), excluding 2 baboons that required euthanasia on day 12 for consumptive coagulopathy. (Black line = without tocilizumab therapy; Red line = with tocilizumab therapy.) Tocilizumab treatment appeared to prevent the sustained histone increase seen after xenotransplantation. (Reprinted with permission from ref. [26]). **b** In vitro histone-induced porcine endothelial cell apoptosis/death is influenced by NF-κB inhibition. The NF-κB inhibitor, parthenolide (at 2 and 8 μM), significantly reduced histone (160 μg/mL)-induced cell apoptosis/death (mean percentage apoptosis/death of 91.4% vs 54%, respectively; both P < 0.05). There was no significant difference in the protective effect of parthenolide at concentrations of 2 and 8 μM (mean percentage apoptosis/death of 54% at both concentrations). (Reprinted with permission from ref. [26])

The direct prothrombotic activity of histone-DNA complexes increases inflammatory cytokine formation, and fosters thrombotic responses by activating TLRs 2, 4, and 9 [48]. Moreover, inflammatory cytokines downregulate thrombomodulin, induce tissue factor, and upregulate plasminogen activator inhibitor [48]. Histones can also cause direct platelet activation [53, 58]. Their levels increase in xenograft recipients when there is evidence for inflammation and coagulation dysfunction [26]. In the absence of IL-6-receptor blockade (with tocilizumab), the mean serum histone level after pig organ transplantation rises significantly [26] (Fig. 3a). A decrease in the number of neutrophils might reduce extracellular histone release [59, 60]. In in vitro studies, histone-induced porcine EC apoptosis/death was significantly reduced by an inhibitor of nuclear factor kappa B (NF-κB), parthenolide (Fig. 3b) [26]. EC apoptosis is observed in many inflammatory and immune disorders [61].

*D-dimer i*s a protein product of cross-linked fibrin degradation. An elevated blood concentration of D-dimer is observed in intravascular coagulation and thrombotic disease [62]. D-dimer may promote the inflammatory cascade by activating neutrophils and monocytes, inducing secretion of inflammatory cytokines (e.g., IL-6) [62–65].

**Fig. 4:**
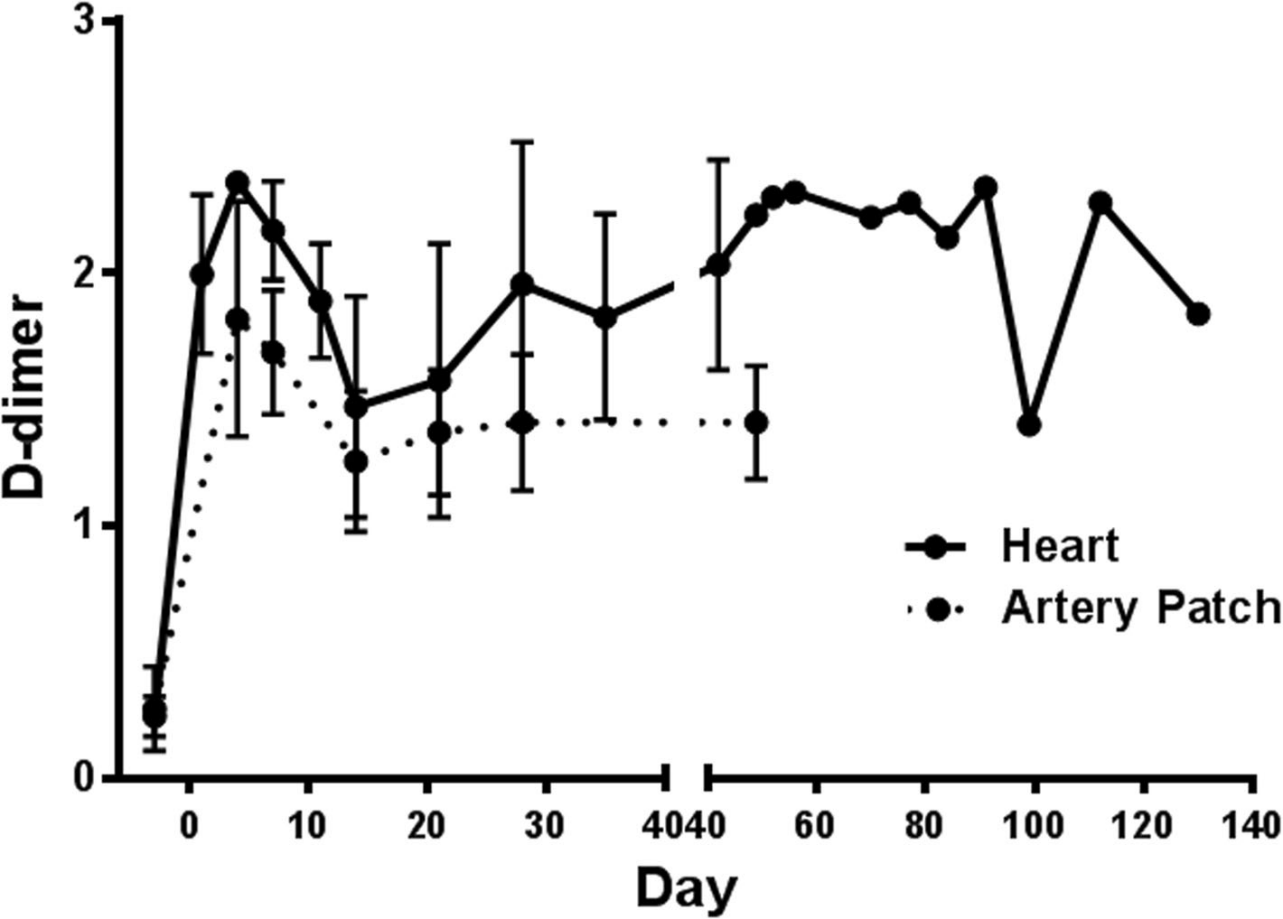
Changes in D-dimer after pig-to-baboon heart (*n* = 4) or artery patch (*n* = 14) transplantation. In heart recipients, mean D-dimer increased from < 0.5μg/ml pre-transplant to > 2.0μg/ml (on post-transplant day 4) and was variable thereafter. In artery patch recipients, mean D-dimer increased from < 0.5μg/ml pre-transplant to 1.4μg/ml on post-transplant day 48 (P < 0.01). (Reprinted with permission from ref. [19])

D-dimer may also be a marker of inflammation [19, 64, 66, 67], and may rise when a xenograft is failing (Fig. 4) [19].

*Pro-inflammatory cytokines/chemokines* help to resist infection, but may induce systemic inflammation [68, 69]. In in vitro studies, porcine IL-6, IL-1β, and TNF-α activated human umbilical vein ECs (HUVECs) [70]. Pig aortic ECs (pAECs) can be significantly activated by human IL-6, IL-17, IL-1β, and TNF-α [70]. For example, (i) human IL-17, IL-1β, and TNF-α increased the expression of adhesion molecule genes (e.g., E-selectin, VCAM-1, and ICAM-1), (ii) human IL-6, IL-17, IL-1β, and TNF-α induced chemokines (e.g., IL-8 and MCP-1) and increased tissue factor expression, and (iii) expression of swine leukocyte antigen (SLA) class-I was induced by human IL-1β and TNF-α [70]. All of the above cytokines/chemokines are likely to promote inflammation and coagulation in response to a xenograft.

**Fig. 5:**
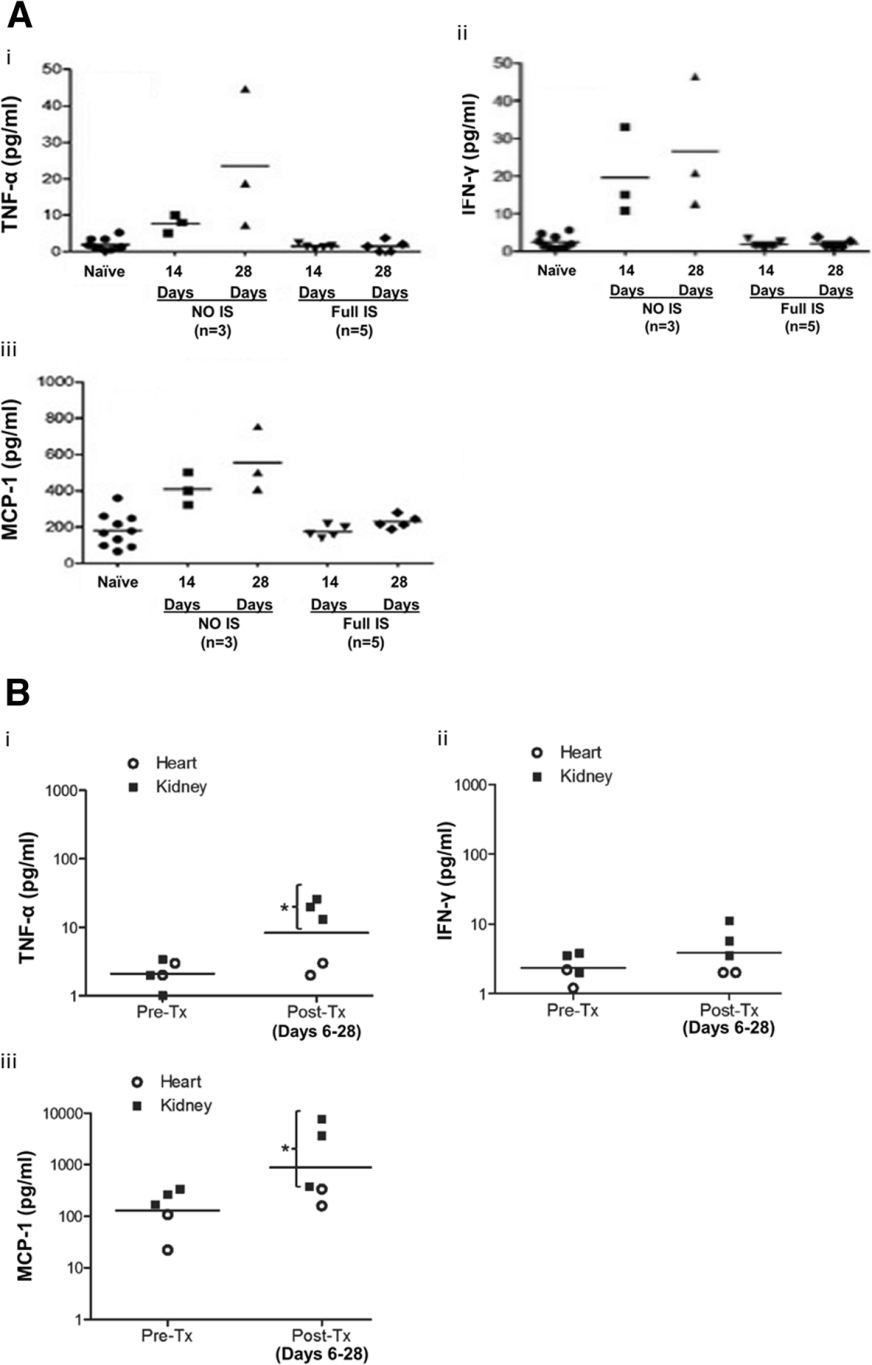
**a** Changes in the levels of selected serum cytokines after pig-to-baboon artery patch transplantation (*n* = 8) (i) TNF-α; (ii) IFN-γ; (iii) MCP-1. In baboons (*n* = 3) that did not receive immunosuppressive therapy after artery patch transplantation, levels of (i) TNF-α, (ii) IFN-γ, and (iii) MCP-1 were significantly higher on post-transplant days 14 and 28 than pre-transplant. When full immunosuppressive therapy was administered to the baboons (*n* = 5), no increase in any of the three cytokines was observed. (Reprinted with permission from ref. [18]). **b** Changes in the levels of selected cytokines after pig-to-baboon kidney (n = 3) or heart (*n* = 2) transplantation. (i) TNF-α; (ii) IFN-γ; (iii) MCP-1. Although the numbers of experiments were small, (i) TNF-α and (iii) MCP-1 were significantly higher in kidney recipients (*P < 0.05), but not in heart recipients. (ii) IFN-γ was not significantly increased in recipients after kidney or heart transplantation. (Reprinted with permission from ref. [18])

In the absence of immunosuppressive therapy, increases in certain cytokine levels are seen after xenotransplantation, but not when immunosuppressive therapy is administered [18] (Fig. 5a,b).

Inflammation plays a key role in *platelet activation and aggregation* [71], which in turn plays an important role in the dysregulation of coagulation seen after xenotransplantation [72]. Extracellular histones bind to TLRs, particularly to TLR2 and TLR4, on platelets, which results in platelet aggregation [51, 53]. In humans, the cytokine, IL-17, can promote platelet activation and aggregation through the ERK2 and P53 signaling pathways [73, 74], although the exact mechanism remains unclear [75]. Recipient platelets might also be activated by binding directly to pig ECs [76]. Human platelets can upregulate tissue factor expression after contact with pAECs in the absence of human serum or antibodies, which can lead to coagulation through thrombin production [77].

There is a relationship between a low *plasma free triiodothyronine (fT3)* and inflammation [78–82]. Plasma fT3 falls following brain death [83, 84], and major surgical procedures, especially heart surgery on cardiopulmonary bypass [85–89].

**Fig. 6:**
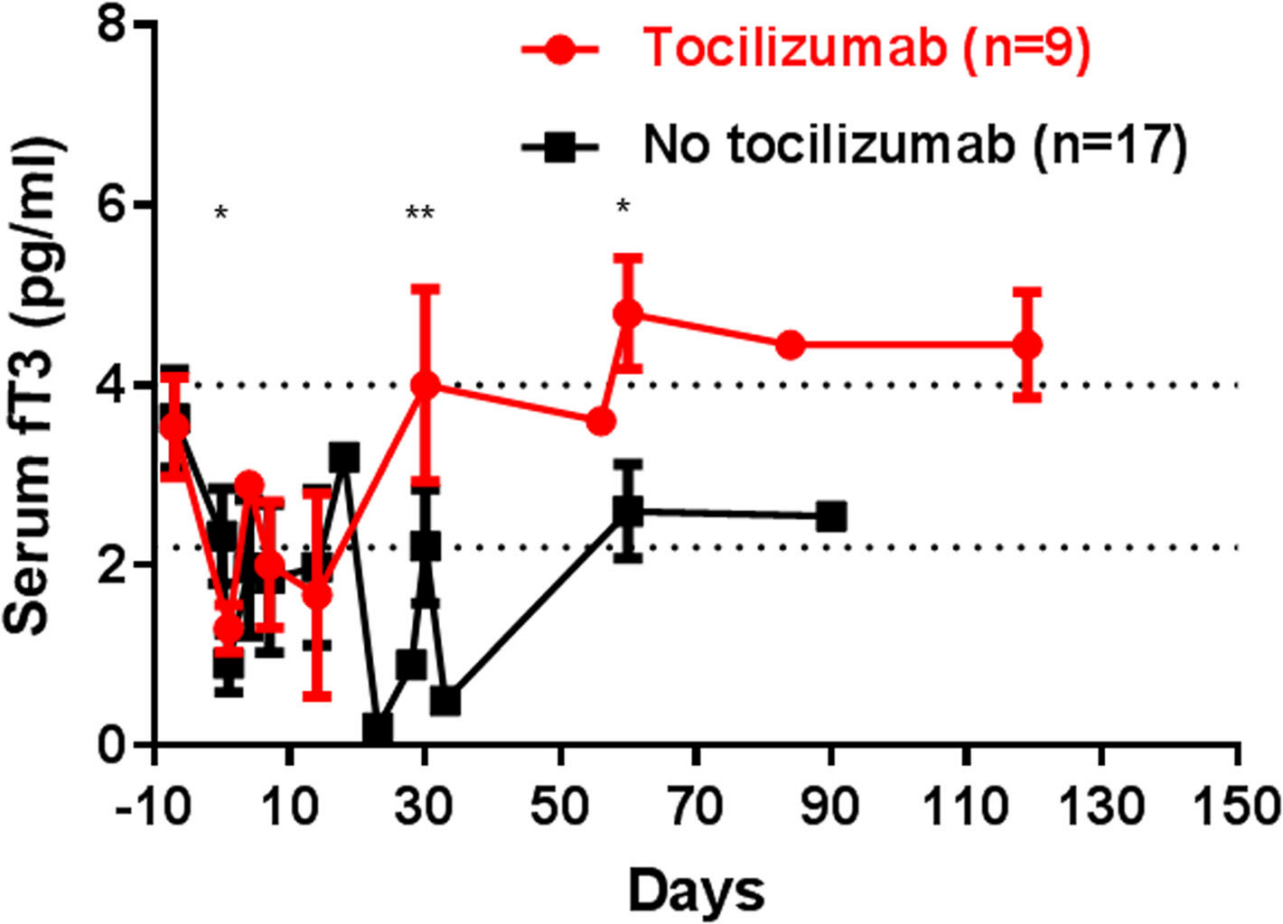
Changes in serum free triiodothyronine (fT3) after pig-to-baboon organ or artery patch transplantation (*n* = 26). The serum fT3 showed an immediate and significant decrease (P < 0.001) in all baboons (n = 26) after pig organ transplantation. In baboons that received the IL-6R blocker, tocilizumab (n = 9), the fT3 recovered more rapidly and to a higher level than in baboons that did not receive tocilizumab (n = 17) (day 1, 1.3 vs 0.9 pg/mL, P < 0.05; day 30, 4.0 vs 2.2 pg/mL, P < 0.01; day 60, 4.8 vs 2.6 pg/mL, P < 0.05, respectively). We concluded that IL-6R blockade reduced inflammation, allowing the fT3 to recover more rapidly. (Reprinted with permission from ref. [26])

In recipient baboons undergoing pig heart, kidney, liver, and artery patch xenotransplants, fT3 falls rapidly, and takes several days to return to pre-transplant levels [26] (Fig. 6). A negative correlation between serum IL-6 and TNF-α with thyroid hormone concentrations has been reported [80]. A persisting low level is almost certainly associated with an inflammatory response to a xenograft [26].

### Evidence for the relationship between inflammation and coagulation in xenograft recipients

Until recently, a major barrier to successful pig organ transplantation in NHPs was dysregulation of coagulation resulting from excessive thrombin generation [90–93]. The activation of thrombin receptors amplifies production of the chemokine, CCL18, and the pulmonary activation-regulated chemokine by mature dendritic cells [94]. Thrombin can upregulate ICAM-1 mRNA and induce ICAM-1 expression on monocytes in vitro [95], and by activating NF-κB [96].

It is well-known that inflammation contributes to activation of coagulation dysfunction [17, 18, 70, 97, 98]. Tissue factor is not only a promoter of thrombin, but also a marker of inflammation [99, 100]. TNF-α [101], IL-6 [102], and C-RP [103] increase tissue factor expression on innate immune cells, which in turn promotes the activation of coagulation [100, 103]. There is an amplification circuit between coagulation and inflammation which results in activation of inflammatory mediators as well as procoagulant factors [20]. Therefore, therapeutic prevention of inflammation may be a major factor in minimizing coagulation dysregulation after pig organ xenotransplantation.

**Fig. 7:**
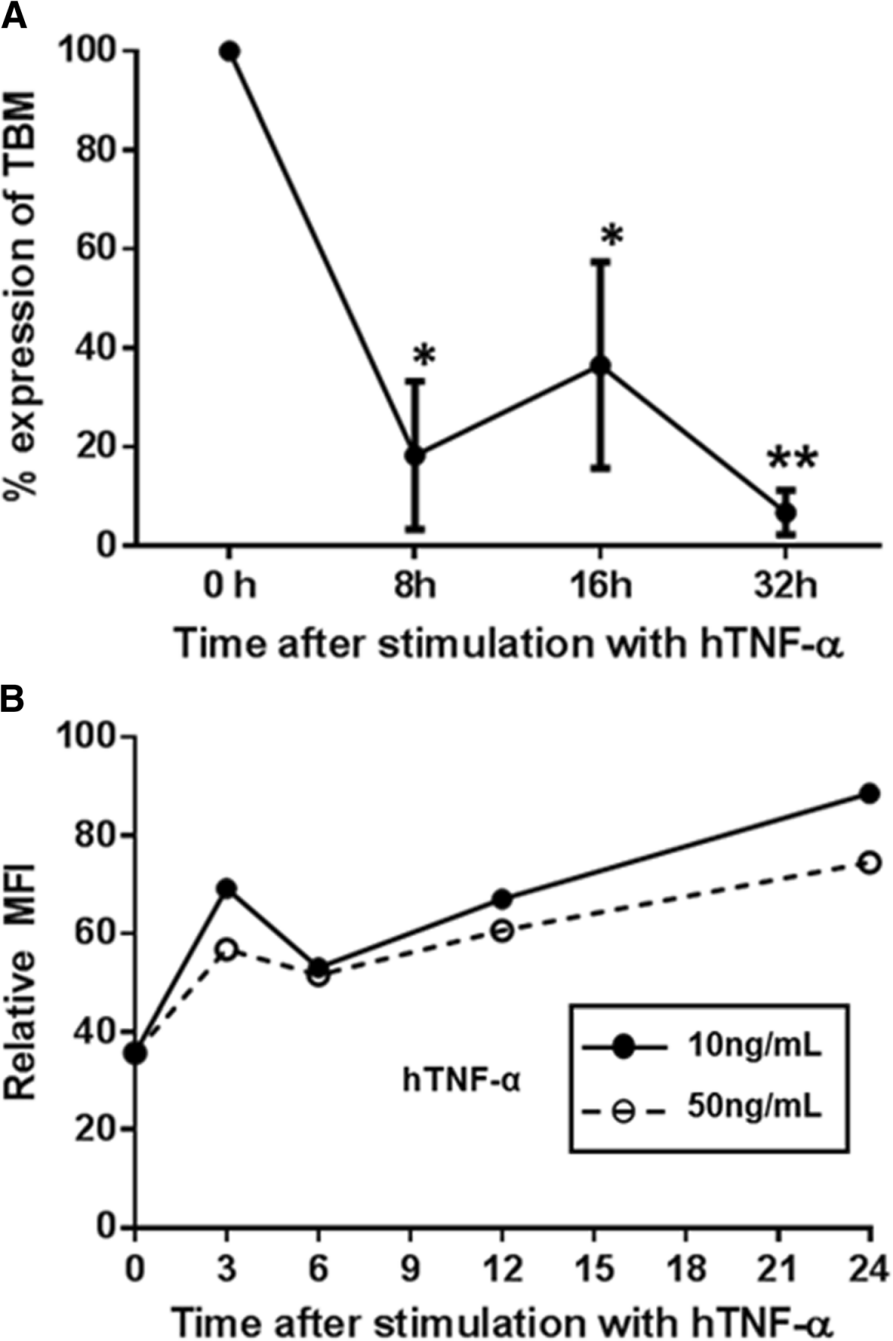
**a** Inflammation down-regulated expression of natural pig thrombomodulin (TBM) Expression of natural pig thrombomodulin was down-regulated after exposure to TNF-α, and was confirmed by real-time PCR (*P < 0.05, **P < 0.01). (The expression of pig thrombomodulin in GTKO/CD46 pig aortic endothelial cells [pAECs] was measured by real-time PCR. The PCR primer sequences used were: pTBM: Sense 5′- GAA GCT ATG AGG TCC AGC CC − 3′; Antisense 5′- CAG ACA GAC AGC GAA GAG CA − 3′.) (Details in ref. [104]). **b** Inflammation did *not* down-regulate expression of transgenic human TBM. The expression of transgenic human thrombomodulin was upregulated, confirmed by flow cytometry. Transgenically-expressed human thrombomodulin would appear to be resistant to down-regulation by inflammation. (The expression of human thrombomodulin in human thrombomodulin-transgenic pAECs was measured by flow cytometry (clone 1A4, BD Biosciences, San Jose, CA)

An important observation made recently indicates that, when pig vascular ECs expressing only natural pig thrombomodulin (which also has an anti-inflammatory effect) are activated by TNF-α, the expression of thrombomodulin is significantly downregulated (Fig. 7a) [98]. This suggests that, when a pig organ is exposed to inflammation (which is *universal* after a pig organ transplant into a NHP), thrombotic microangiopathy is likely to develop. The absence of the anti-inflammatory effect of human thrombomodulin may result in the early development of consumptive coagulopathy [6]. In contrast, *transgenic* expression of human thrombomodulin is *not* downregulated, thus maintaining both its anticoagulant and anti-inflammatory effects (Fig. 7b) [98].

### Evidence for the relationship between inflammation and the immune response in xenograft recipients

The significant increase in certain cytokines/chemokines after xenotransplantation likely results from innate immune cell activity, and may well be a causative factor in xenograft injury [17, 18]. Inflammation and the innate immune response augment the adaptive immune response [70, 98]. Systemic upregulation of inflammatory markers is related to inefficient blockade of the T cell-dependent adaptive immune response [105].

**Fig. 8:**
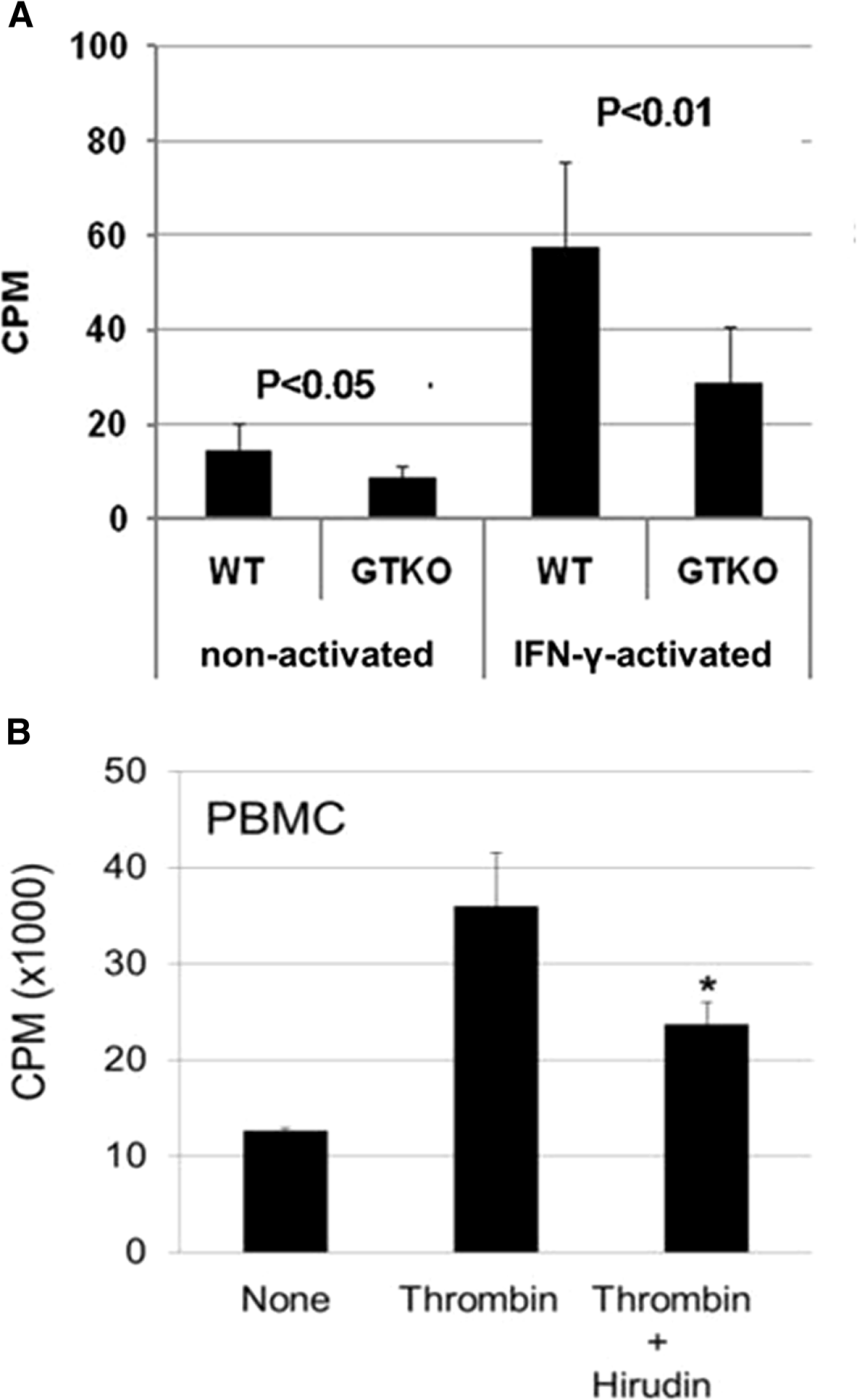
**a** IFN- γ-activation increases the proliferative response of human peripheral blood mononuclear cells (PBMCs) to wild-type (WT) and GTKO pig aortic endothelial cells (pAECs). When non-activated, the proliferative response to WT pAECs was greater than to GTKO pAECs (P < 0.05). There was an increase in the PBMC response when the pAECs were activated by IFN-γ, the response to WT pAECs again being significantly greater than to GTKO pAECs (P < 0.01) . The study illustrates how inflammation can increase the immune response to a xenograft. (CPM = counts per minute; SI = stimulation index). (Reproduced with permission from ref. [106]). **b** Thrombin activates T cell proliferation. The degree of activation of GTKO pig PBMCs by thrombin was comparable to that resulting from stimulation of the cells by porcine interferon-gamma (pIFN-γ). Thrombin-stimulated activation of the human cellular response was reduced by the addition of hirudin, confirming that thrombin was the stimulatory factor. (Reproduced with permission from ref. [97])

In an in vitro study, there was a significant increase in the human peripheral blood mononuclear cell (PBMC) proliferative response when pAECs were activated by pig IFN-γ, supporting the concept that inflammation augments the immune response to a xenograft [106] (Fig. 8a). The induction of T cell tolerance after transplantation is inhibited by inflammation [25]. By affecting the immune response, cytokine and chemokine secretions influence the outcome of allotransplantation [107, 108]. Increased IL-7, IL-8, and IFN-γ-induced protein 10, chemokine ligand 9, and chemokine ligands 2 and 5 are associated with early allograft dysfunction [109–111].

Inflammation, coagulation, and the immune response have a complex inter-relationship [23, 50]. For example, thrombin activates the human cellular response to pig cells in vitro, and induces a T cell proliferative response to the same extent as IFN-γ activation (Fig. 8b) [97].

### Potential strategies to prevent inflammation in xenotransplantation recipients

Several strategies aimed at preventing or reducing excessive inflammation after xenotransplantation have been tested, some of which are clinically-approved.

#### Drug therapy (Table 2)

**Table 2:** Anti-inflammatory agents that may prevent or reduce SIXR

Agent	Results of therapy		References
Anti-complement agents	IL-6, IL-8, MCP-1	↓	[13]
IL-6 receptor blockade	fT3, IL-6, MCP-1	↑	[6, 13, 19, 26, 30]
	Histones, C-RP, SAA	↓	[19, 26]
IL-6 inhibitor	C-RP	↓	[112]
TNF-α inhibitor	E-selectin, VCAM-1	↓	[113]
NF-κB inhibitor (parthenolide)	Platelet aggregation, endothelial cell apoptosis	↓	[26, 114]
Alpha-1 antitrypsin (AAT)	IL-8, IL-1β, TNF-α	↓	[115]
Platelet inhibitor (aspirin)	IL-6	↓	[116]
T_3_	Adenosine phosphate, creatine phosphate, lactate	↓	[88, 117, 118]
	Glycogen	↑	
	TNF-α, IL-6	↓	[119]

#### Corticosteroids

Corticosteroids activate several genes, including inhibitors of NF-*κ*B, which has an anti-inflammatory effect [120]. After their administration to pig heart xenograft recipients, the levels of IL-6, IL-8, and MCP-1 were reduced [13]. However, D-dimer remained increased, irrespective of corticosteroids and/or anti-inflammatory therapy, suggesting that an inflammatory response persisted [13].

#### Anti-complement agents

Although cobra venom factor (CVF) is primarily administered to deplete complement [121], MCP-1, IL-8, and IL-6 are reduced after its administration [13]. After cobra venom factor administration in baboons with pig artery patch grafts, IL-6, IL-8, and MCP-1 remained lower than, or comparable to, pre-transplant levels [13]. Eculizumab is an anti-C5 humanized monoclonal antibody and inhibits the terminal complement effector pathway by preventing its cleavage by the C5 convertase [122]. It modifies the cytokine profile by increasing IFN-γ and IL-17 and lowering IL-4. [123–125]. Cp40, a cyclic 14-amino acid peptide, is a complement inhibitor that inhibits the generation of pro-inflammatory effectors (e.g., TNF-α, IL-1β, and IL-17) through inhibiting the activation of C3 [126, 127]. C1-inhibitor is the only known plasma protein inhibitor of serine proteases, C1s and C1r, of the classical complement pathway. It decreases some pro-inflammatory cytokines (TNF-α*,* IL-18) and increases a protective cytokine (IL-10) [128, 129].

#### IL-6 receptor blockade, and IL-6 inhibitors

Treatment of the NHP recipient of a pig xenograft with the IL-6 receptor blockade agent, tocilizumab, results in greatly decreased levels of C-RP (Fig. 1a) [19] and serum histones (Fig. 3a) [26]. However, D-dimer remained elevated (Fig. 4) [13, 19]. Blockade of IL-6 receptors is also associated with more rapid recovery of the fall in the level of fT3 seen after xenotransplantation (Fig. 6) [26].

Tocilizumab has several other beneficial effects on the immune response to a graft. It reduces the number of memory B cells [130, 131]) and plasma cells [132], but increases regulatory B cells [133], and the ratio of regulatory T cells [134]. It also reduces monocytes and myeloid dendritic cells [135]. Recipients of kidney allografts treated with tocilizumab suffer less antibody-mediated rejection [136], and have reduced donor-specific antibody levels [137].

However, recent evidence indicates that tocilizumab, although binding to primate IL-6 receptors, does *not* bind to IL-6 receptors on the pig graft [70], and therefore may have no protective effect on the graft. The IL-6 inhibitor, siltuximab, has a therapeutic effect in Castleman disease and certain inflammatory diseases by neutralizing IL-6 production [138]. IL-6 neutralization with siltuximab resulted in sustained C-RP suppression in Castleman disease [112], but it is not completely effective in xenotransplantation [Zhang G, et al., manuscript in preparation].

#### Anti-histone antibodies

Extracellular histones and TLR pathways are major targets for treating a variety of inflammatory conditions. Anti-histone therapy has the potential to prevent histone-induced inflammation in xenotransplantation [26]. The administration of an anti-histone antibody (e.g., anti-histone H4 monoclonal antibody) inhibits cytokine production and has a protective effect on various inflammatory injuries [45, 56, 139–147]. The protective effects of rTBM against histone toxicity are mediated through both activated protein C-dependent and -independent ways [148]. Anti-histone antibodies have not yet been tested in in vivo models of xenotransplantation.

#### TNF-α inhibitors

EC activation is reduced by a TNF-α inhibitor [113]. A TNF-receptor fusion protein (TNF-RFP) has reduced inflammation in an in vivo xenoperfusion model, although the mechanism of its function is poorly understood [113].

#### NF-κB inhibitors

NF-κB plays a crucial role in enhancing the cellular responses to inflammation. Thrombin not only activates NF-κB, but also upregulates NF-κB-dependent genes [87]. As extracellular histones induce expression of tissue factor on ECs potentially through the NF-κB pathway, this amplifies thrombin generation [149]. The NF-κB inhibitor, parthenolide, reduced porcine EC apoptosis/death in vitro [26] (Fig. 3b). Parthenolide has also been reported to reduce endotoxic shock and prevent inflammation in immune glomerulonephritis [150]. It is used as prophylactic treatment for migraine, and has been reported to have a beneficial effect in clinical trials [151].

#### Alpha 1-antitrypsin (AAT)

AAT, a prototypic serine protease inhibitor, is abundant in human blood. Although mainly produced by hepatocytes [152], it is also produced by other cells (e.g., epithelial cells [153], monocytes [154], macrophages and neutrophils [155, 156], intestinal epithelial cells [157], alpha and delta cells of human pancreatic islets [158], and cancer cells [159]). Plasma levels of AAT increase during inflammation and infection [160].

AAT has anti-inflammatory, anti-leukocyte migratory, anti-apoptotic, and anti-thrombotic effects [161–166]. Treatment with AAT significantly decreases the levels of pro-inflammatory cytokines (IL-8, IL-1β, TNF-α) [115]. In monkeys with islet allotransplants, AAT prevented an inflammatory response [167] but, when baboons received artery patch grafts from genetically-engineered pigs, treatment with AAT had no effect on IL-8 and C-RP levels [13].

#### Platelet inhibitors

Aspirin is widely used as a preventative against vascular disease, and is associated with a reduction in myocardial infarction and stroke [168]. In addition, there is evidence that aspirin down-regulates some proinflammatory cytokines (e.g., IL-6) [116] and proinflammatory signaling pathways, including NF-κB [169–171].

#### Triiodothyronine (T3)

It remains uncertain whether, in the presence of a pig xenograft, the administration of T_3_ can suppress the inflammatory state [79], but T3 treatment reduces inflammatory cytokines (e.g., TNF-α, IL-6), improving glycemic control in diabetic rats [119]. Nevertheless, as there is a fall in fT3 in all baboons following pig organ transplantation [30], we have found it beneficial to administer T3 to increase fT3 levels.

#### Genetic modification of the organ-source pig (Table 3)

**Table 3:** Genetic modifications of the organ-source pig that may be protective against the inflammatory response

Genes	Function	References
Hemeoxygenase-1 (HO-1)	anti-inflammatory, anti-apoptotic	[14, 172–178]
A20 (tumor necrosis factor-α-induced protein)	anti-inflammatory, anti-apoptotic	[179–181]
Thrombomodulin (TBM)	anticoagulation, anti-inflammatory	[182–187]
Endothelial protein C receptor (EPCR)	anticoagulation, anti-inflammatory	[188]
Ectonucleoside triphosphate diphosphohydrolase-1 (CD39)	anticoagulation, anti-inflammatory	[189–191]
Tissue factor pathway inhibitor (TFPI)	anticoagulation, anti-inflammatory	[192, 193]

#### Expression of hemeoxygenase-1 (HO-1)

**Fig. 9:**
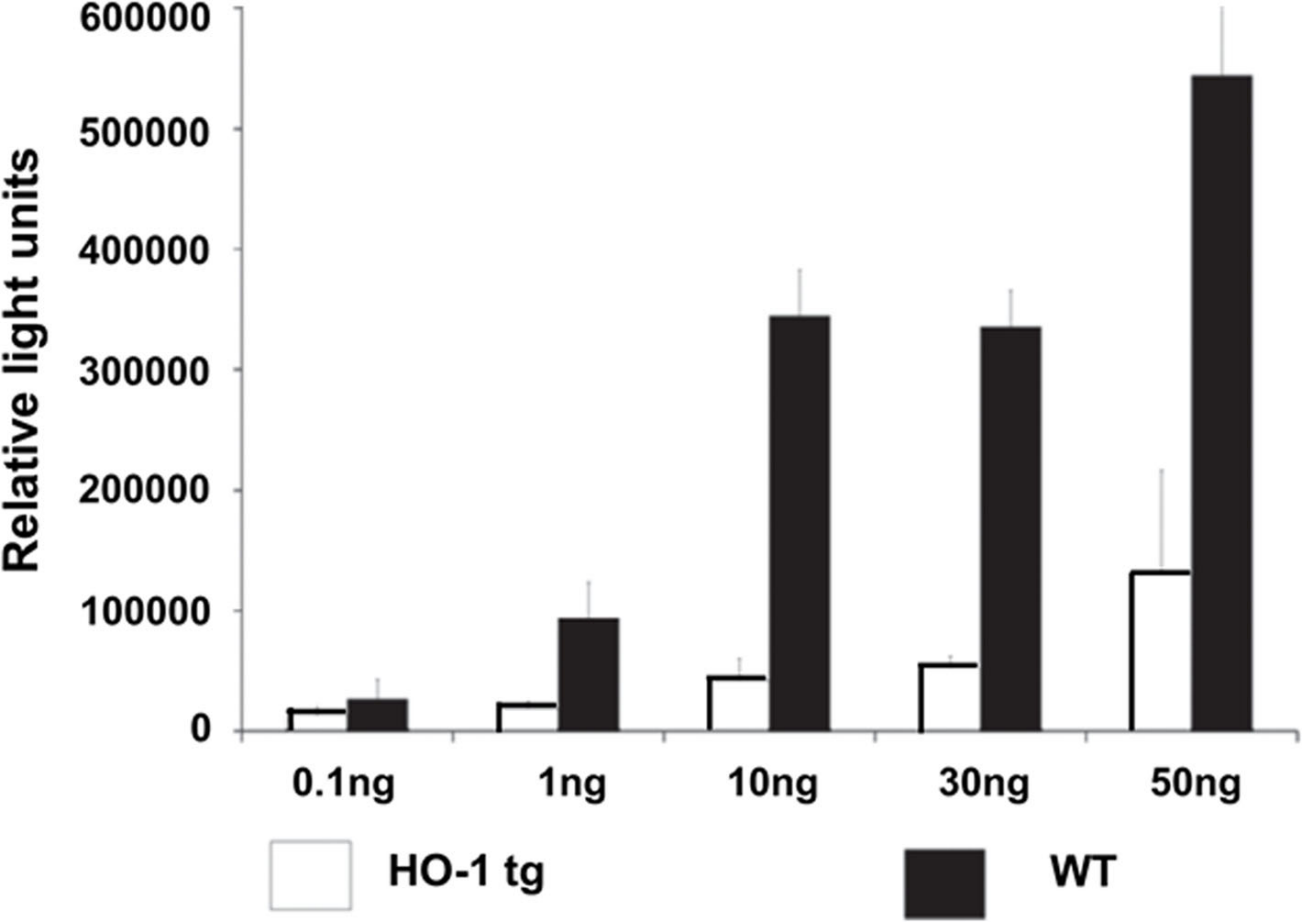
TNF-α-induced apoptosis was reduced by transgenic expression of hHO-1. Human hemeoxygenase-1 (hHO-1) transgenic pig aortic endothelial cells (pAECs) were protected against TNF-α-mediated apoptosis, measured by a caspase 3/7 assay. pAECs from hHO-1 transgenic pigs were better protected against TNF-α-mediated apoptosis compared to WT pAECs. (Modified from ref. [175])

HO-1 is known to have an anti-inflammatory effect and reduces cell apoptosis [14, 172–178]. It is an anti-oxidant enzyme, which is regulated by the erythroid 2-related factor 2 (Nrf2) pathway [194]. The activation of HO-1 can prevent TNF-α-induced inflammatory and oxidative damage by up-regulating the Nrf2/HO-1 signaling pathway [195]. hHO-1 expression on porcine cells prevents TNFα- and cycloheximide-mediated apoptosis (Fig. 9) [173–176], and results in the downregulation of adhesion molecules, e.g., E-selectin, ICAM-1, and VCAM-1 [175]. Organs expressing hHO-1 were shown to be critical for prolonged survival of mouse cardiac xenografts in rats [173, 177], and expression of hHO-1 in pig islets prolonged their survival in mice, and decreased immune cell infiltration and islet cell apoptosis [178].

#### Expression of A20

A20, a TNF-α-induced protein, has been shown to be anti-inflammatory and anti-apoptotic [179–181]. A20 is an important regulator of inflammatory signaling, which counteracts NF-κB activation. Several reports suggested that A20 plays a crucial role in inhibiting NF-κB signaling in response to TNF-α and microbial products [180, 181]. pAECs from hA20 transgenic pigs underwent significantly reduced apoptosis compared to wild-type pAECs [179]. hA20-transgenic pig hearts were partially protected against ischemia/reperfusion injury [179].

#### Expression of coagulation-regulatory proteins

Several coagulation-regulatory proteins have anti-inflammatory properties, e.g., thrombomodulin [182–187], endothelial protein C receptor (EPCR) [188], ectonucleoside triphosphate diphosphohydrolase-1 (CD39) [189–191], and tissue factor pathway inhibitor (TFPI) [192, 193]. The N-terminal lectin–like domain of thrombomodulin was reported to possess direct anti-inflammatory activity and to suppress complement activation [182]. Thrombomodulin also has anti-inflammatory effects through its capacity to promote generation of activated protein C [183–186], which exerts anticoagulant activity and has a direct cytoprotective effect [196]. Endothelial protein C receptor also elicits activated protein C-dependent and -independent anti-inflammatory effects [188]. CD39 is a major vascular nucleoside triphosphate diphosphohydrolase, and converts adenosine triphosphate (ATP), and adenosine diphosphate (ADP) to adenosine. CD39 was demonstrated to protect kidney grafts from ischemia-reperfusion injury via anti-inflammatory adenosine receptor signaling [189], and to protect islets from the instant blood-mediated inflammatory reaction (IBMIR) [190]. TFPI is an essential anticoagulant protein that acts by preventing the activation of the blood coagulation proteases, factor VII to VIIa (fVIIa) and factor X to Xa (fXa) [197]. In murine pneumococcal pneumonia, recombinant human TFPI reduces IL-6, TNF-α, MCP-1, IFN-γ, keratinocyte-derived cytokine, and macrophage-inflammatory protein-2, and increases the anti-inflammatory cytokine IL-10 [192].

## Conclusions

Systemic inflammation may be playing a crucial role in pig organ xenotransplantation through activating the coagulation cascade and immune response. The administration of anti-inflammatory agents or the genetic modification of the organ-source pig by the introduction of human inflammation-regulatory transgenes may be beneficial to prevent or control inflammation. Control of inflammation is likely to allow a reduction in the intensity of exogenous immunosuppressive therapy. If immunological tolerance to a xenograft is to be obtained, then control of inflammation may be essential.

## Abbreviations

AAT: Alpha 1-antitrypsin
C-RP: C-reactive protein
EC: Endothelial cell
fT_3_: Free triiodothyronine
HO-1: Hemeoxygenase-1
IL: Interleukin
MCP-1: Monocyte chemotactic protein-1
NF-kB: Nuclear factor kappa B
NHP: Nonhuman primate
SAA: Serum amyloid A
SIXR: Systemic inflammation in xenograft recipients
TFPI: Tissue factor pathway inhibitor
TLR: Toll-like receptor
TNF-α: Tumor necrosis factor-alpha
